# Evaluation of effectiveness of granulocyte-macrophage colony-stimulating factor therapy to cancer patients after chemotherapy: a meta-analysis

**DOI:** 10.18632/oncotarget.24890

**Published:** 2018-06-15

**Authors:** Wen-Liang Yu, Zi-Chun Hua

**Affiliations:** ^1^ The State Key Laboratory of Quality Research in Chinese Medicine, Macau Institute for Applied Research in Medicine and Health, Macau University of Science and Technology, Macao, China; ^2^ The State Key Laboratory of Pharmaceutical Biotechnology, School of Life Sciences, Nanjing University, Nanjing, China; ^3^ Changzhou High-Tech Research Institute of Nanjing University and Jiangsu TargetPharma Laboratories Inc., Changzhou, China

**Keywords:** GM-CSF, hematologic index, meta-analysis

## Abstract

The impact of granulocyte-macrophage colony stimulating factor (GM-CSF) on hematologic indexes and complications remains existing contradictory evidence in cancer patients after treatment of chemotherapy. Eligible studies up to March 2017 were searched and reviewed from PubMed and Wanfang databases. Totally 1043 cancer patients from 15 studies were included in our research. The result indicated that GM-CSF could significantly improve white blood cells count (SMD = 1.16, 95% CI: 0.71 – 1.61, Z = 5.03, *P* < 0.00001) and reduce the time to leukopenia recovery (SMD = -0.85, 95% CI: -1.16 – -0.54, Z = 5.38, *P* < 0.00001) in cancer patients after treatment of chemotherapy. It also could improve absolute neutrophil count (SMD = 1.11, 95% CI: 0.39 – 1.82, Z = 3.04, *P* = 0.002) and significantly shorten the time to neutropenia recovery (SMD = -1.47, 95% CI: -2.20 – -1.75, Z = 3.99, *P* < 0.0001). However, GM-CSF could not improve blood platelet (SMD = 0.46, 95% CI: -0.37 – -1.29, Z = 1.10, *P* = 0.27). And GM-CSF had significant connection with fever (RR = 3.44, 95% CI: 1.43 – 8.28, Z = 2.76, *P* = 0.006). The publication bias existed in the data of the impact of GM-CSF on blood platelet and complication. In conclusions, GM-CSF had an intimate association with some hematologic indexes and complications. Our study suggested that more hematological indexes and even more other indexes need to be observed in future studies.

## INTRODUCTION

Chemotherapy has been widely used for treating different cancers for many years. Whereas chemotherapy can lead to many adverse drug reactions, such as hematologic toxicity, thromboembolism, and neurotoxicity. [1–3]. Hematologic toxicity is a common adverse reaction such as neutropenia and leukopenia. It not only delays the time of next therapy but also leads to the life-threatening events (such as severe infection, bleeding, copper deficiency and protein malnutrition) if decreased blood cells had not been managed timely [4, 5]. The previous studies had confirmed that chemotherapy-induced neutropenia or leukopenia could reduce survival rates in patients with advanced cancer [3, 6]. Thus, the hematologic indexes, including white blood cells (WBC) count, absolute neutrophil count (ANC), blood platelet (PLT) count and monocytes count, are important objective indexes in cancer patients after chemotherapy.

GM-CSF is the cytokine most extensively used as hemopoietin in the clinical practice, it can promote the activation, proliferation, and differentiation of myeloid precursor cells in the body. Beside GM-CSF plays an important role in the recruitment, development, and maturation of dendritic cells, which are necessary for the subsequent T helper cell type 1 and cytotoxic T lymphocyte activation. GM-CSF also can enhance the function of mature granulocytes and mononuclear phagocytes [5, 7–9]. In the 1990s, GM-CSF was approved by Food and Drug Administration (FDA) as a drug for treatment of older adults with acute myeloid leukemia, after induction chemotherapy, to shorten the time to neutrophil recovery and to reduce the incidence of life-threatening infections. Thus GM-CSF is often used to treat chemotherapy-induced neutropenia and leucopenia [8, 10]. GM-CSF was also approved by FDA for myeloid reconstitution following allogeneic bone marrow transplantation (BMT), autologous BMT or peripheral blood stem cell transplantation. Furthermore, GM-CSF was approved for peripheral blood stem cell mobilization, BMT failure, and engraftment delay. GM-CSF also has many pro-inflammatory functions [11]. The trade name of GM-CSF includes sargramostim, leucomax (American name), “Te Li Er”, “Ge Ning” (Chinese name), and molgramostim (Indian name).

GM-CSF has been used to treat chemotherapy-included neutropenia and leucopenia for many years, and many different clinical responses and data are accumulated. However, these clinical data and responses, including the impact of GM-CSF on WBC count, ANC, BLT, and complications, still lack systematic analysis and evaluation. Hence this study aims to quantify the data from previous clinical studies via meta-analysis, for systematically analyzing and evaluating the clinical impact of GM-CSF on hematologic indexes and complications.

## RESULTS

### Research results and quality assessment

**Figure 1:**
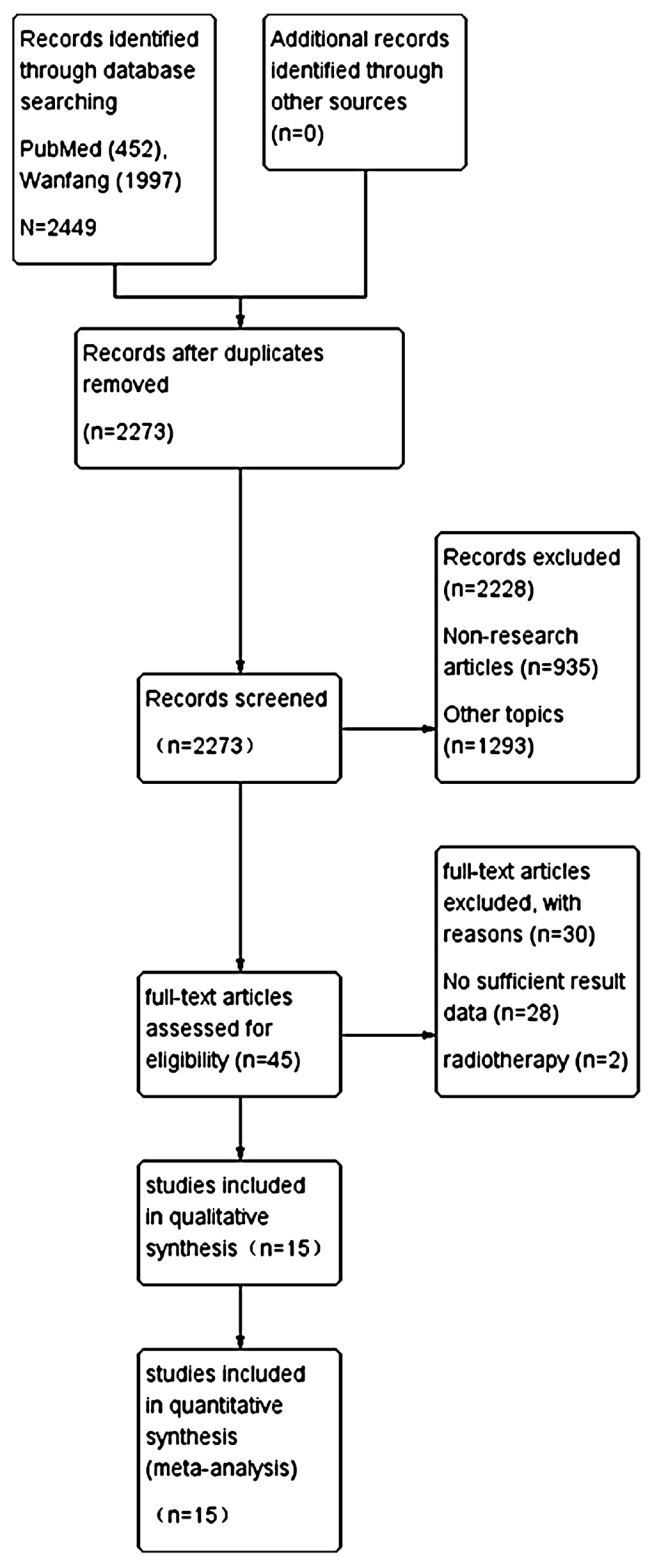
Flow-diagram of the literature selection process

The process of the literature search was included in a PRISMA flow diagram (Figure 1). The preliminary screening identified 2449 potentially relevant publications by reviewing titles and abstracts, including 176 duplicates, 935 non-research article, and 1293 irrelevant studies. And then we screened the full-texts of the remaining 45 articles, 28 and 2 papers were excluded with insufficient result data and radiotherapy, respectively. Finally, 15 studies [12–26] (Table 1) were included in our research, including 5 English papers [12–16] and 10 Chinese papers [17–26]. The quality assessment of 15 studies was shown in Table 2. The agreement for selection of studies between two authors was high.

**Table 1:** Basic characteristics of studies included in this meta-analysis

Study publish year	Country/race	Type of study	Treatment details	No. of patient	Sex ratio (male%)	Age	Weight (kg)	KPS	Sources of medication	Dosage and *duration* of GM-CSF	Clinical response	Toxic and side effects
B Kopf *et al.* 2006	Italy/Europe	clinical trial	CT	29	48	21-56	—	—	—	1 time per 1d (5 mg/kg)	87.5%	Y
Nathan L *et al*. 1999	Canada/North America	clinical trial	CT	20	0	31-69	—	—	America	1 time per 1d (250μg/m^2^)	82.9%	N
Montero *et al.* 2005	America/North America	clinical trial	CT	23	—	31-69	—	70-90	America	1 time per 1d (250μg/m^2^)	96%	Y
Stephen E *et al.* 1996	America/North America	clinical trial	CT	142	0	25-69	46-133	≥80	America	1 time per 1d (250μg/m^2^)	97%	Y
A. Le Cesne *et al.* 2000	France/ Europe	clinical trial	CT	294	42	19-76	—	≥70	Germany	1 time per 1d (250μg/m^2^)	21.3%	Y
He *et al.* 2001	China/Asia	clinical trial	CT	50	64	8-77	—	—	China	1 time per 1d (75μg/d)	92%	Y
Zhou *et al.* 1999	China/Asia	clinical trial	CT	60	63	16-68	40-80	—	China	1 time per 1d (5μg/kg)	86.7%	Y
Sun 2005	China/Asia	clinical trial	CT	28	71	24-71	—	≥60	China	1 time per 1d (150μg/d)	92.9%	Y
Liu *et al.* 2000	China/Asia	clinical trial	CT	55	60	14-70	—	≥60	China	1 time per 1d (5μg/kg)	73.7%	N
Chen *et al.* 2003	China/Asia	clinical trial	CT	50	58	23-71	—	>60	China	1 time per 1d (150μg/d)	—	Y
Liu *et al.* 2000	China/Asia	clinical trial	CT	78	54	23-78	—	>60	China	1 time per 1d (75μg/d)	—	Y
Zhou *et al.* 1999	China/Asia	clinical trial	CT	60	60	16-70	40-95	70-100	China	1 time per 1d (5μg/kg)	—	Y
Ji 2015	China/Asia	clinical trial	CT	60	50	25-68	—	—	China	1 time per 1d (100μg/d)	—	N
Yuan *et al.* 2002	China/Asia	clinical trial	CT	30	67	10-69	—	—	China	1 time per 1d (3-5μg/kg)	93.33%	Y
Zhou *et al.* 1999	China/Asia	clinical trial	CT	56	60	17-67	45-84	—	China	1 time per 1d (5μg/kg)	—	Y

Notes: CT: chemotherapy. KPS: Karnofsky Performance Status. Y: the article mentions toxic and side effects. N: the article do not have or do not mention toxic and side effects.

**Table 2:** Reporting quality of 15 randomized control trials based on the CONSORT 2010 Checklist [n (%)]

Section/topic	No	Checklist item	1^a^	0^b^	NI^c^	Remark
**Title and abstract**						
	1a	Identification as a randomized trial in the title	5 (33.3)	10 (66.7)		
	1b	Structured summary of trial design, methods, results, and conclusions (for specific guidance see CONSORT for abstracts)	10 (66.7)	5 (33.3)		
**Introduction**						
Background and objectives	2a	Scientific background and explanation of rationale	8(53.3)	2(13.3)	5(33.3)	
	2b	Specific objectives or hypotheses	10 (66.7)	3 (20.0)	1 (6.7)	
**Methods**						
Trial design	3a	Description of trial design (such as parallel, factorial) including allocation ratio	15 (100)	0 (0.0)		
	3b	Important changes to methods after trial commencement (such as eligibility criteria), with reasons	0 (0.0)	0 (0.0)		unable to judge
Participants	4a	Eligibility criteria for participants	13 (86.7)	0 (0.0)	2 (13.3)	
	4b	Settings and locations where the data were collected	2 (13.3)	13 (86.7)		
Interventions	5	The interventions for each group with sufficient details to allow replication, including how and when they were actually administered	15 (100)	0 (0.0)		
Outcomes	6a	Completely defined prespecified primary and secondary outcome measures, including how and when they were assessed	0 (0.0)	15 (100)		
	6b	Any changes to trial outcomes after the trial commenced, with reasons	3 (20.0)	12 (80.0)		
Sample size	7a	How sample size was determined	14 (93.3)	1 (6.7)		
	7b	When applicable, explanation of any interim analyses and stopping guidelines	5 (33.3)	10 (66.7)		
Sequence generation	8a	Method used to generate the random allocation sequence	9(60.0)	6(40.0)		
	8b	Type of randomization; details of any restriction (such as blocking and block size)	8(53.3)	7(46.7)		
Allocation concealment mechanism	9	Mechanism used to implement the random allocation sequence (such as sequentially numbered containers), describing any steps taken to conceal the sequence until interventions were assigned	7(46.7)	8(53.3)		
Implementation	10	Who generated the random allocation sequence, who enrolled participants, and who assigned participants to interventions	0 (0.0)	15 (100)		
Blinding	11a	If done, who was blinded after assignment to interventions (for example, participants, care providers, those assessing outcomes) and how	2 (13.3)	13 (86.7)		
	11b	If relevant, description of the similarity of interventions	0 (0.0)	15 (100)		
Statistical methods	12a	Statistical methods used to compare groups for primary and secondary outcomes	9(60.0)	6(40.0)		
	12b	Methods for additional analyses, such as subgroup analyses and adjusted analyses	4 (26.7)	11 (73.3)		
**Results**						
Participant flow (a diagram is strongly recommended)	13a	For each group, the numbers of participants who were randomly assigned, received intended treatment, and were analyzed for the primary outcome	15 (100)	0 (0.0)		
	13b	For each group, losses and exclusions after randomization, together with reasons	0 (0.0)	15 (100)		
Recruitment	14a	Dates defining the periods of recruitment and follow-up	0 (0.0)	14 (93.3)	1 (6.7)	
	14b	Why the trial ended or was stopped	5 (33.3)	10 (66.7)		
Baseline data	15	A table showing baseline demographic and clinical characteristics for each group	11 (73.3)	3 (20.0)	1 (6.7)	
Numbers analyzed	16	For each group, number of participants (denominator) included in each analysis and whether the analysis was by original assigned groups	15 (100)	0 (0.0)		
Outcomes and estimation	17a	For each primary and secondary outcome, results for each group, and the estimated effect size and its precision (such as 95% confidence interval)	13 (86.7)	0 (0.0)	2 (13.3)	
	17b	For binary outcomes, presentation of both absolute and relative effect sizes is recommended	0 (0.0)	15 (100)		
Ancillary analyses	18	Results of any other analyses performed, including subgroup analyses and adjusted analyses, distinguishing pre-specified from exploratory	0 (0.0)	15 (100)		
Harms	19	Important harms or unintended effects in each group (for specific guidance see CONSORT for harms)	14 (93.3)	1 (6.7)		
**Discussion**						
Limitations	20	Trial limitations, addressing sources of potential bias, imprecision, and, if relevant, multiplicity of analyses	6(40.0)	9(60.0)		
Generalizability	21	Generalizability (external validity, applicability) of the trial findings	13 (86.7)	2 (13.3)		
Interpretation	22	Interpretation consistent with results, balancing benefits and harms, and considering other relevant evidence	7(46.7)	4(26.7)	4(26.7)	
**Other information**						
Registration	23	Registration number and name of trial registry	0 (0.0)	15 (100)		
Protocol	24	Where the full trial protocol can be accessed, if available	0 (0.0)	15 (100)		
Funding	25	Sources of funding and other support (such as supply of drugs), role of funders	3 (20.0)	12 (80.0)		

Notes: check measure as 1^a^: reported; 0^b^: not reported; NI^c^: partially reported, but insufficient. STRICTA: Standards for Reporting Interventions in Clinical Trials of Acupuncture. RCTs: Randomized controlled trials.

### Impact of GM-CSF on WBC count in cancer patients

**Figure 2:**
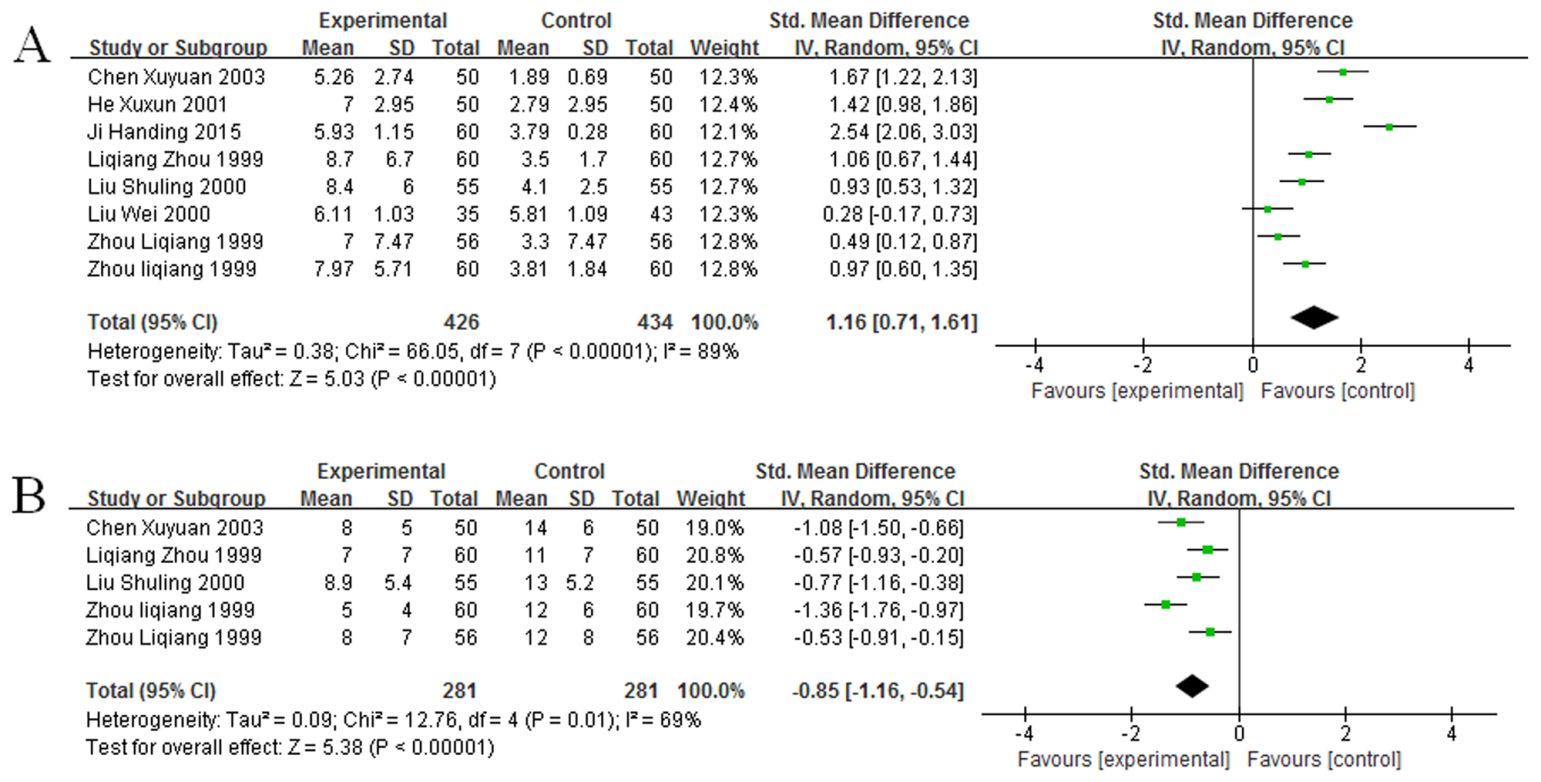
Forest plot showing the connection between GM-CSF and WBC count **(A)** Impact of GM-CSF on WBC count. **(B)** Impact of GM-CSF on the recovery time of leukopenia.

Eight studies [17, 18, 20–24, 26] which included 469 cancer patients after chemotherapy reported the association between GM-CSF and WBC count. Homogeneity test showed that these studies had not homogeneity (χ^2^ = 66.05, *P* < 0.00001, I^2^ = 89%, Figure 2A). Thus we used the random-effect model to make statistics. And the result demonstrated that GM-CSF was associated with increased WBC count (SMD = 1.16, 95% CI: 0.71 – 1.61, Z = 5.03, *P* < 0.00001, Figure 2A). Furthermore, we analyzed the connections between GM-CSF and the time of leukopenia. Five studies [18, 20, 21, 23, 26], included 281 patients, showed the data regarding the effect of GM-CSF on the time to neutropenia. And these studies had not homogeneity as well (χ^2^ = 12.76, *P* = 0.01, I^2^ = 69%, Figure 2B). Thus, the data were calculated by the random-effect model. The result demonstrated that GM-CSF shortened the time to WBC count recovery (SMD = -0.85, 95% CI: -1.16 – -0.54, Z = 5.38, *P* < 0.00001, Figure 2B), thus GM-CSF could made the WBC count recovery more quickly.

### Impact of GM-CSF on ANC in cancer patients

**Figure 3:**
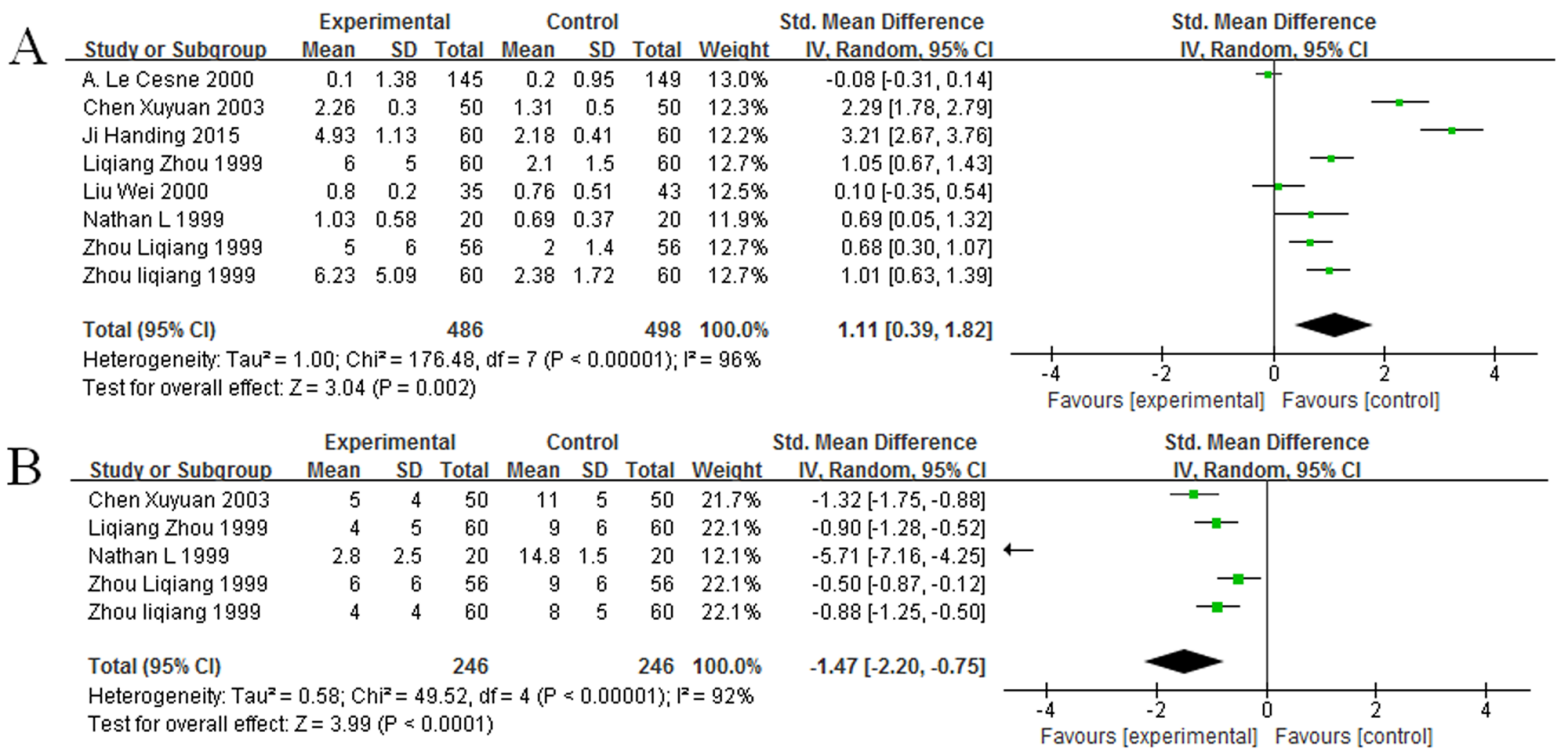
Forest plot showing the connection between GM-CSF and ANC **(A)** Impact of GM-CSF on ANC count. **(B)** Impact of GM-CSF on the recovery time of neutropenia.

Eight studies [13, 16, 18, 21–24, 26] provided the data regarding ANC, which included 533 cancer patients after chemotherapy. All studies had significant heterogeneity (χ^2^ = 176.48, *P* < 0.00001, I^2^ = 96%, Figure 3A). Thus, random-effect model was used to calculate them, and the result suggested that GM-CSF was connected with increased ANC (SMD = 1.11, 95% CI: 0.39 – 1.82, Z = 3.04, *P* = 0.002, Figure 3A). And then, five studies [13, 18, 21, 23, 26] reported the data concerning the time to neutropenia recovery, which included 246 patients. Homogeneity test revealed not homogeneity (χ^2^= 49.52, *P* < 0.00001, I^2^ = 92%, Figure 3B), these data were calculated by using random-effect model. The result confirmed that GM-CSF could shorten the time to ANC recovery (SMD = -1.47, 95% CI: -2.20 – -1.75, Z = 3.99, *P* < 0.0001, Figure 3B).

### Impact of GM-CSF on PLT count in cancer patients

**Figure 4:**
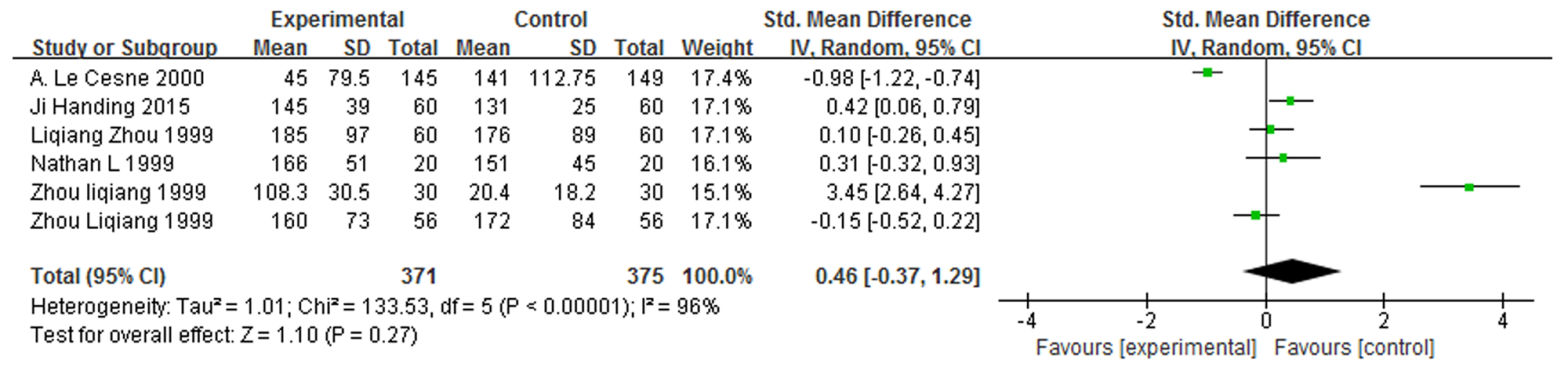
Forest plot showing the connection between GM-CSF and PLT count

Six studies [13, 16, 18, 24–26] including 375 cancer patients after chemotherapy, offered the data concerning PLT count. Homogeneity test showed all studies were heterogeneous (χ^2^ = 133.53, *P* < 0.00001, I^2^ = 96%, Figure 4). Thus we used random-effect model to calculate them. The result showed that the association between GM-CSF and PLT count does not exist (SMD = 0.46, 95% CI: -0.37 – -1.29, Z = 1.10, *P* = 0.27, Figure 4).

### Complications

**Figure 5:**
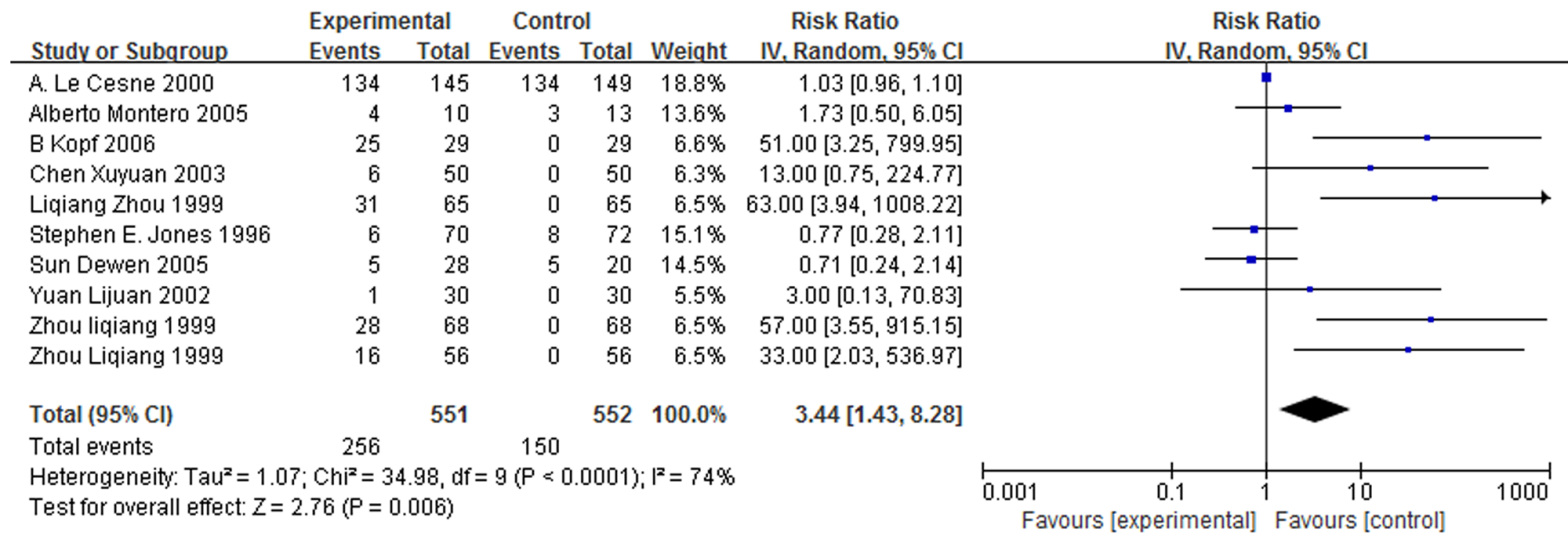
Forest plot showing the connection between GM-CSF and fever Notes: Invalid lines vertical horizontal scale is 1. Each horizontal line represents the upper and lower bounds of the 95% confidence interval of the study. The length of the line represents the range of confidence interval. The green or blue solid in the center of a horizontal line is the positions of OR value or RR value, and the size of solid represents the weight of the study. Black diamond represents the effect quantity and confidence interval of multiple studies merging.

Because of individual differences, different patients often had different degrees of complications after treatment with GM-CSF, such as fever, local reactions, bone and muscle pain. Except for fever, most of the complications could be tolerated and do not lead to serious consequences. Ten studies [12, 14–19, 21, 23, 25, 26] provide the data of fever, and 805 patients were included. Homogeneity test showed that there is no homogeneity in these studies (χ^2^ = 34.98, *P* < 0.0001, I^2^ = 74%, Figure 5). Hence random-effect model was used to calculate these data. The results suggested that GM-CSF had significant connection with fever (RR = 3.44, 95% CI: 1.43 – 8.28, Z = 2.76, *P* = 0.006, Figure 5).

### Publication bias

**Figure 6:**
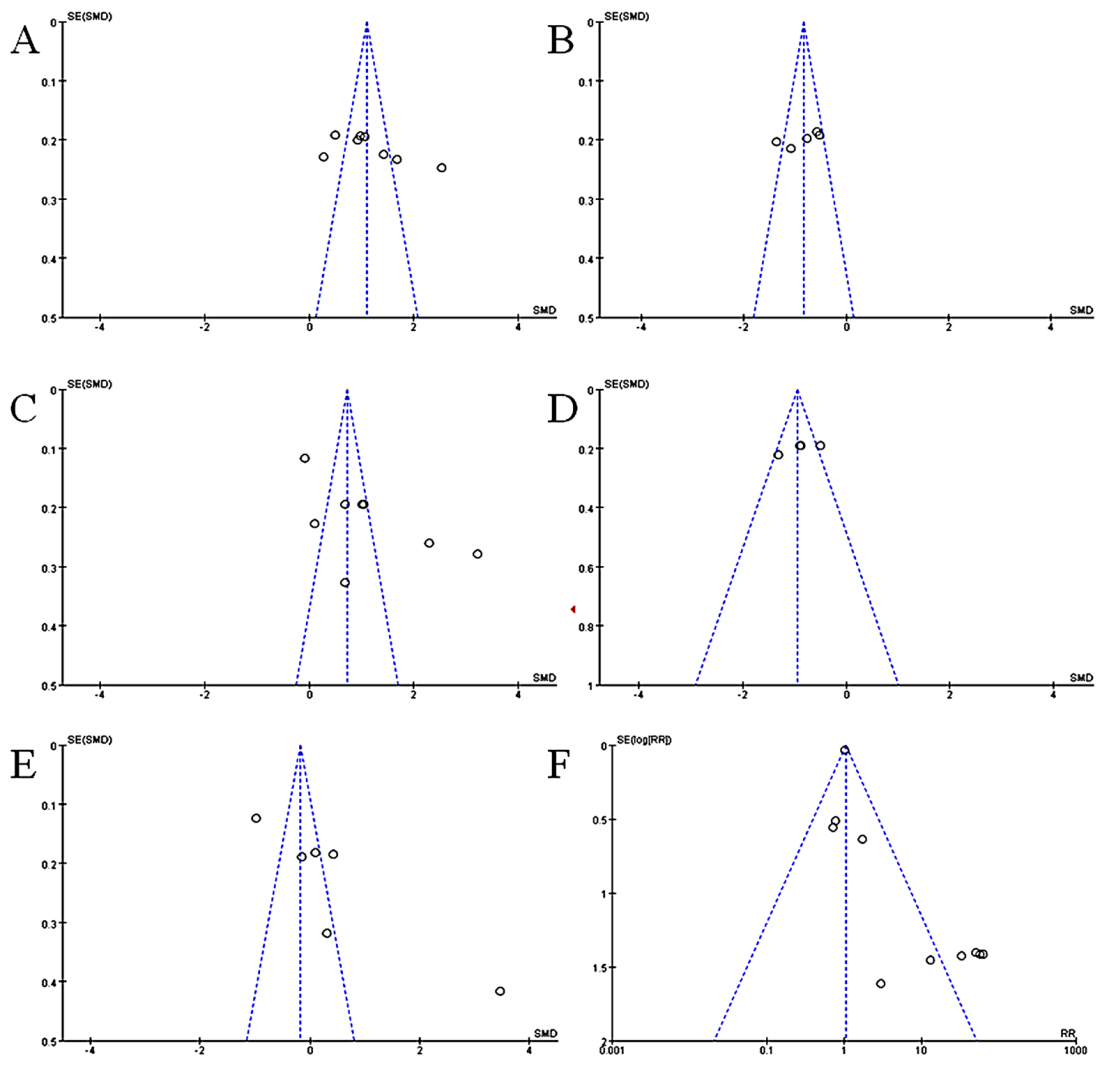
Funnel plot for publication bias **(A)** Impact of GM-CSF on WBC count. **(B)** Impact of GM-CSF on the time of leukopenia. **(C)** Impact of GM-CSF on ANC. **(D)** Impact of GM-CSF on the time of neutropenia. **(E)** Impact of GM-CSF on PLT count. **(F)** Impact of GM-CSF on fever.

As shown in Figure 6, the funnel plots for evaluating the publication bias for the impact of GM-CSF on hematologic index and complications. Open circles represent studies included in the meta-analysis. The perpendicular in the center indicates the summary proportion. And the other two dotted lines represent the 95% CI. On the visual assessment of funnel plot, there is no evidence of publication bias was revealed on the association between GM-CSF with WBC count, the time to neutropenia recovery and leucopenia recovery (Figure 6A, 6B and 6D). However, the publication bias existed on the studies regarding the impact of GM-CSF on ANC, PLT count, and complications.

## DISCUSSION

Recent studies regarding chemotherapeutic agents show that targeted therapies have received increasing attention [27–30]. However chemotherapy-included hematologic toxicity still exists. It not only increases pain to the patient, but also reduce medication adherence, delay the treatment cycle, and even lead to life-threatening events. Thus it is important for the patient to control hematologic toxicity after treatment with chemotherapy.

Over the past decade, many types of research focused on the relationship between cytokines and chemotherapy-included hematologic toxicity [31–36]. Since GM-CSF and granulocyte colony stimulating factor (G-CSF) were approved by FDA in the 1990s, they were both widely used to treat various types of cancer patients with chemotherapy-induced neutropenia and leucopenia, and achieved an excellent result. Because of the outstanding clinical efficacy of GM-CSF and G-CSF, they have made hundreds of millions of dollars each year in sales. G-CSF, as a member of cytokine families, is often used to promote the production of granulocytes or antigen presenting cells. And the clinical efficacy of G-CSF has been systematically evaluated and analyzed in many studies [10, 37–39]. However, as a cytokine that has a similar efficacy to G-CSF, the clinical efficacy of GM-CSF is lack of systematic analysis and evaluation. Thus this study fills the gap. The result of our study confirmed that GM-CSF can help cancer patient after treatment of chemotherapy quickly improving WBC count and ANC. And GM-CSF has no association with PLT count, however, the result regarding GM-CSF and PLT from A. Le Cesne et al. [16] were that GM-CSF could significantly improve the PLT count in cancer patients. Besides, GM-CSF has a significant connection with fever.

Although the included data come from different countries, including Italia, Canada, France, America, and China. The drugs (GM-CSF) that they used were manufactured in different places and even have different trade names. The types of patients were all cancer patients after treatment of chemotherapy, administration route were all intravenous injection, administration time was all at least 24 hours after treatment of chemotherapy, and clinical observation index was all hematologic index. moreover, the clinical response rate in these studies was similar (73.7%-97%) except A. Le Cesne et al. [16] (21.3%). Besides, three studies were published by one first author Zhou LQ [18, 23, 26], which means that Zhou was a specialist in this field. And these three studies used three different brands of GM-CSF to treat different patients at the same dosage and duration. It not only avoided the difference in the research system but also studied drug standardization of GM-CSF.

Our study also exists some limitations, the main limitations are sample size and the publication time of studies. According to the data needs of this study, the result of the search was so. The method and the duration of GM-CSF administration still keep the same. The publication bias is not robust. And the data of statistical analysis were expressed in median and range. That was the reason why we did this study. We tried to find the connections between GM-CSF and hematologic index by analyzing available data, which can help the doctor better observe the efficacy of GM-CSF at the treatment stage, rather than waiting until the end of treatment.

The potential heterogeneity come from patients, there are over 30 cancer types with the total patients number, including lung cancer, breast cancer, lymphoma etc. And they were treated with different chemotherapy. However, firstly when GM-CSF is clinically used for chemotherapy-induced neutropenia and leucopenia, it is a broad-spectrum drug that does not target a particular tumor. GM-CSF is used simultaneously with chemotherapy drugs. The rapid proliferation and differentiation of myeloid precursor cells are sensitive to chemotherapeutic drugs, thus affecting the efficacy of chemotherapy. Secondly, the mechanism of GM-CSF therapy is actually immunotherapy. Thus the heterogeneity in immunity of patients is more important than cancer types. Unfortunately the data included in our meta-analysis lack the immunity information of patients.

In conclusion, according to the results of this study, we found that an included study [16] shown a significant difference from the other studies in two results: the impact of GM-CSF on PLT count and the clinical response rate of GM-CSF, respectively. The most likely reason for this is sources of medication, however, we cannot rule out other possible causes. Thus we believed that only the index of WBC count and ANC were used to judge the outcome of GM-CSF treatment in previous studies, which may not completely reflect the efficacy. We suggested that hematological indexes need to be expanded in future studies. Further, the other indexes should also be observed, such as immunological indexes.

## MATERIALS AND METHODS

### Search strategy

Firstly we searched the systematic reviews and meta-analysis on the role of GM-CSF in cancer patient after treatment of chemotherapy. No reviews or meta-analysis were found. Secondly, we searched the PubMed and Wanfang databases for studies reporting the use of GM-CSF in cancer patient after treatment of chemotherapy. And the following keywords were used in the process of search, respectively: GM-CSF cancer therapy clinic, GM-CSF white blood cell, sargramostim, leucomax and molgramostim. The languages of articles were limited in English and Chinese. “Full text” and “Human” were used to filter article. Finally, the reference lists of primary studies were reviewed by two authors. The latest search happened on March 9, 2017.

### Selection criteria for considering studies for this review

The patients from included studies were diagnosed with various forms of cancer, and all of them received systemic chemotherapy. At the same time, they agreed to participate in an experimental study of the impact of GM-CSF on hematologic indexes and complications. Then we excluded the following studies: (1) editorials, abstract and letters, (2) studies in which the clinical outcome was not reported, (3) single-patient case report or short case series involving fewer than four patients (4) multiple publications from the same study to prevent erroneous patient count and (5) graduation theses.

### Initial review of studies

The initial database was compiled, and all duplicate articles were eliminated. We screened these citations depending on title, abstract and the relevant studies for inclusion based on the criteria identified previously. Only after assessment of the full-text articles by two authors, the studies were finally selected for inclusion in the review. Any disagreement was resolved by discussion between two authors.

### Data abstraction

The data of initial review were recorded on a standard data extraction form by both the authors independently. The following items were extracted: (1) publication details (title, authors, years and other citation details, including geographic locale); (2) type of study (clinical trial or review); (3) treatment details, (chemotherapy or radiotherapy); (4) patients details (number, sex ratio, age, weight, KPS); (5) dosage and duration of GM-CSF; (6) sources of medication; and (7) clinical response and toxic or side effects, if any.

### Assessment of study quality

The CONSORT 2010 checklist (25 items) was used to assess the reporting quality of eligible studies. According to this checklist, two authors assessed the reporting quality, respectively. Every 25 items had one common criterion, including “yes”, “no” and “I”. They represented “adequately reported”, “Not reported” and “partially reported, but insufficient”, respectively. Besides, “unable to judge” represented if there was lacking information regarding the item. Any disagreement was resolved by discussion between the two authors.

### Statistical analysis

Cochran's *Q* test and Higgins’ I^2^ statistics were used to making homogeneity test for eligible studies. A *P*-value ≤ 0.1 and/or I^2^ ≥ 50% indicated significant heterogeneity, the data should be calculated by the random-effect model. Accordingly A *P*-value > 0.1 and/or I^2^ < 50% indicated significant homogeneity, the data should be calculated by fixed-effect model [40]. Standard mean differences (SMD) and pooled risk ratios (RR) with 95% confidence intervals (95% CI) were used to evaluate the association of GM-CSF with hematologic indexes and complications, respectively. The Z test was used in effect comparison between experimental and control. A *P*-value < 0.05 in the Z test for pooled RR, or no overlap of the 95% CI with 1 was considered statistically significant. And the publication bias was assessed by visual inspection of funnel plots. All data analyses were performed by using Review Manager 5.3 (Cochrane Collaboration, London, UK) and adhered to the PRISMA guidelines.
